# Diarrheal disease outbreak in Gaidatar village of Rautahat District, Nepal

**DOI:** 10.1186/s13104-019-4156-9

**Published:** 2019-03-08

**Authors:** Kul Raj Rai, Reena Kiran Mukhiya, Santosh Thapa, Ganesh Rai, Sabina KC, Phanu Maya Thapa, Prasha Shrestha, Shiba Kumar Rai

**Affiliations:** 1 ShiGan International College of Science and Technology (SICOST), Narayangopal, Chowk, Chakrapath, Kathmandu, Nepal; 2 Present Address: CAS Key Laboratory of Pathogenic Microbiology and Immunology, Institute of Microbiology, Chinese Academy of Sciences (CAS), Beijing, China; 3 Department of Pathology and Immunology, Baylor College of Medicine, Houston, Texas USA; 4 Texas Children’s Microbiome Center, Department of Pathology, Texas Children’s Hospital, Houston, Texas USA

**Keywords:** Diarrhea, Cholera, *Vibrio cholerae*, Nepal

## Abstract

**Objective:**

Diarrheal diseases, including cholera, remain a major public health concern in developing countries like Nepal. This study investigated a diarrheal outbreak that affected over 1500 people in Gaidatar village of Rautahat district in central Nepal and sought to identify the source and causation of the disease. Stool samples were collected from individuals with acute diarrheal illness (n = 16) and healthy non-diarrheal children (n = 39), along with samples from local drinking water sources (n = 8) and their sewage system (n = 10). None of the individuals were sampled multiple times. Diarrheic stool and sewage samples were analysed for the presence of *Vibrio cholerae*, while coliforms were tested in drinking water samples following standard microbiological protocols. Enteric parasites were tested in both diarrheic and non-diarrheic stool samples.

**Results:**

*Vibrio cholerae* O1 Ogawa serotype was isolated in 18.7% of the diarrheic stool and 20.0% of the sewage. Coliforms were found in all drinking water samples, with 87.5% testing positive for fecal coliform. Additionally, 43.6% of the stool samples (n = 55) had at least one of the intestinal parasites tested, primarily *Giardia lamblia* (21.8%). However, almost all parasites were found in non-diarrheal stool. Taken together, our results provide evidence that the diarrheal outbreak was associated with *V. cholera*e O1 Ogawa serotype, possibly transmitted through the drinking water sources contaminated with fecal matters from their sewage (drainage) system. These findings warrant regular surveillance of drinking water sources to help prevent future outbreaks.

## Introduction

Diarrheal diseases still remain a major public health threat for developing countries and is attributed to poor sanitation, unhygienic practices and inadequate supply of purified drinking water [1]. Diarrhea, transmitted via fecal–oral route, is caused by various pathogens, including bacteria, viruses, protozoa, and helminths. Of the diarrheal diseases, cholera (an acute diarrheal illness caused by the bacterium *Vibrio cholerae*), alone is responsible for an annual 1.3–4.0 million morbidity and 21,000–143,000 mortality worldwide [2, 3]. A key epidemiological feature of cholera is its ability to cause outbreaks that can quickly lead to devastating epidemics, if not controlled [4]. Among many serogroups of *V. cholerae*, two clinically indistinguishable groups-O1 and -O139 are responsible for the global cholera outbreaks [3].

The number of cholera cases reported to WHO by its member countries has increased in recent years. In 2016, 38 countries reported a total of 132,121 cholera cases (out of which 54%, 32% and 13% were from Africa, Hispaniola and Asia, respectively), including 2420 deaths [3]. A descriptive analysis of cholera outbreaks during 2003–2012 demonstrated that more than two-third of the outbreaks occurred in Southeast Asia, including Nepal [5]. However, underreporting of the cholera cases is a major challenge for the control of the disease and underreported cholera cases have accounted for a significant number of deaths. These discrepancies in the number of cases reported versus the number that actually occur are often resultant from poor surveillance systems and inconsistency in case definition and reporting systems among and within countries. For example, as highlighted in a recent report, the annual number of cholera cases reported to the WHO by the Indian government was several times lower than the actual number of cases [6].

In the context of Nepal, diarrheal diseases are a major public health threat [7] and have been ranked second in the national list of research priorities [8]. Specifically, in regards to cholera, it has been endemic in Nepal for many years as evidenced by several large outbreaks previously documented in and around Kathmandu and other regions of the country [9]. The largest cholera outbreak occurred in Jajarkot and its neighboring districts in 2009. This outbreak affected ~ 30,000 people and led to more than 500 deaths [10]. Cholera outbreak due to drug resistant *V. cholerae* serogroup O1 biotype El Tor serotype Ogawa has also been reported in Nepal [11] and *V. cholerae* has also been detected in 43.5% of sewerage/river system in Kathmandu valley [12]. With this background, the present study reports the findings of a diarrheal outbreak (June 2014) investigated in Gaidatar village of Rautahat District in central Nepal.

## Main text

### Methods

#### Ethics statement

Ethical approval for the study was obtained from the Institutional Review Committee of ShiGan Health Foundation (Kathmandu, Nepal). Before collecting samples, informed consent was obtained from the adult patients and from guardians on behalf of all participants under the age of 16 years.

#### Study area

Gaidatar village is located about 55 km north of Gaur, the administrative headquarter of Rautahat district (about 7 km from Chandranigahpur Bazaar in the East–West Highway) in Terai region of central Nepal. The outbreak of diarrheal disease occurred in June 2014. Upon approval of the concerned authorities, a team of microbiologists visited the village to collect samples for investigations. The visit was coordinated by the concerned governmental health authorities both at the central and local levels.

#### Sample collection and processing

Following informed consent, stool samples were collected from 16 patients visiting a health facility at Gaidatar for treatment of acute diarrheal illness and from 39 non-diarrheal healthy school children living in the same village. A total of 55 stool samples (diarrheal = 16, non-diarrheal = 39) were individually collected in sterile screw-capped plastic containers. None of the individuals were sampled multiple times. In addition, sewage samples from their drainage system (n = 10) were collected using Moore’s technique [12, 13] to investigate possible source of contamination. Furthermore, water samples from the local drinking water sources (n = 8) were also collected for bacteriological investigation and directly placed into the Colilert test system (IDEXX Laboratory, Tokyo, Japan). About 7000 people belonging to ~ 1200 households of Gaidatar ward numbers 3 and 4 use the water as their principal source of drinking water (personal communication, Ward Office of Gaidatar 3 and 4). 0.5–1 g of diarrheic stool samples and the sewage samples were subjected to enrichment in alkaline peptone water (pH 8.6). The remaining diarrheal stool samples were fixed in 10% formal-saline along with non-diarrheal stool for detection of enteric parasites. All samples were transported to the laboratory of ShiGan International College of Science and Technology in Kathmandu maintaining a cold chain. The samples were processed and analysed for the presence of *V. cholerae*, coliforms, and enteric parasites following standard microbiological protocols.

#### Isolation and identification of *V. cholerae*

Diarrheic stool samples (n = 16) and sewage samples (n = 10) enriched in alkaline peptone water were inoculated on TCBS (thiosulfate-citrate-bile salts-sucrose) agar and incubated at 37 °C for 15 h. *V. cholerae* like colonies on TCBS agar were subjected for identification by biotyping and serotyping using polyvalent *V. cholerae* O1 and monovalent (Inaba and Ogawa) antisera (Denka Seiken Co. Ltd, Tokyo, Japan).

#### Detection of fecal indicator bacteria in drinking water

Eight water samples collected from the local drinking water sources were processed using Colilert system (IDEXX Laboratory) following manufacturer’s recommendations. The Colilert test system consists of a tube with dehydrated media containing two indicators: ortho-nitro-phenyl-galactoside (ONPG) and 4-methylumbelliferyl-β-d-glucuronide (MUG). After an overnight incubation at 37 °C, total coliform bacilli produced yellow color, while *Escherichia coli* demonstrated bluish fluorescence color upon exposure to UV light (365 nm).

#### Detection of parasites in stool

A total of 55 stool samples (diarrheal = 16, non-diarrheal = 39) fixed in 10% formal-saline were concentrated by formal-ether sedimentation method [14] and examined under the microscope (40×) for the presence of intestinal parasites.

#### Statistical analysis

Data was analysed in Microsoft Excel and Chi square test was used for the statistical analysis.

### Results

#### *V. cholerae* in stool and sewage

Among the total 16 stool samples collected from 16 individuals (one sample/person) with acute diarrheic illness and 10 environmental (sewage) samples, 18.7% (3/16) and 20.0% (2/10) yielded *V. cholerae*, respectively (Table 1). The 3 *V. cholerae* positive diarrheal samples were from 3 different individuals. Altogether, 19.2% of the samples (fecal and sewage) subjected for alkaline peptone water enrichment showed growth of *V. cholerae*. All isolates were identified as *V. cholerae* O1 Ogawa serotype.

**Table 1:** *Vibrio cholerae* isolated from diarrheic stool and sewage samples

Sample type	Source	No. of samples	No. of *V. cholerae* positive samples in TCBS (%)
Diarrheic stool	Patients	16	3 (18.7)
Sewage	Environment	10	2 (20.0)
Total		26	5 (19.2)

#### Fecal indicator bacteria (FIB) in drinking water

All eight of the drinking water samples collected from different points of the village were positive for coliform bacilli (100%) and of them, 87.5% (7/8) were positive for fecal coliform bacilli (*Esch. coli*).

#### Enteric parasites in fecal samples

Of the total 55 formal-saline fixed stool samples (both diarrheal and non-diarrheal), 43.6% (24/55) were positive for at least one of enteric parasites tested (Table 2). Parasite positive rate was significantly higher among school children when compared to the patients with acute diarrheal illness (p = 0.013).

**Table 2:** Detection of enteric parasites in formal-saline fixed stool samples

Sample type	Source	No. of samples	No. of parasite positive samples (%)	p-value
Diarrheic stool	Patients	16	1 (6.2)	0.013
Non-diarrheic stool	School children (healthy)	39	23 (60.0)	
Total		55	24 (43.6)	

Among the parasite positive samples, 70.8% (17/24) had single parasite while the remaining samples (29.2%) contained more than one parasite. Protozoan parasites were the predominant one. Hookworm was the only helminthic parasite detected. As described in Table 3, *Giardia lamblia* was the most dominant, followed by *Entamoeba coli* and *E. histolytica* and others.

**Table 3:** Frequency of individual organism in parasite positive stool samples

Type of parasites	Frequency	% (out of 31)
Protozoa: *Giardia lamblia*	15	48.4
*Entamoeba coli*	7	22.6
*E. histolytica*	4	12.9
*Endolimax nana*	2	6.4
Helminth: Hookworm	3	9.6

### Discussion

With the start of monsoon season in June 2014, an outbreak of acute diarrheal illness with abdominal pain occurred in Gaidatar village (about 7 km from Chandranigahapur Bazaar located at East–West Highway) in Rautahat District of central Nepal. The outbreak affected over 1500 people. When we arrived at the site, some of the patients had already left the local health facility/camp with prescribed medicine. Hence, we were able to collect stool samples only from 16 patients undergoing treatment at the health facility.

Growth positivity for *V. cholera*e in the present study (18.7% of diarrheic stool samples) was lower when compared to the previous outbreak findings in Nepal (26.7–41.3%) as reported in various studies [15–19]. The three *V. cholerae* positive diarrheic stool samples were collected from three different patients who had been taking antibiotics for 1–2 days prior to sample collection. However, the majority of the patients had a longer history of antibiotic use (at least 1 week). Thus, the low *V. cholerae* positive rate in the diarrheic stool samples in this study is probably due to the fact that samples were collected after the patients had already started antibiotic therapy.

We were able to isolate *V. cholerae* in 20.0% of the sewage (environmental) samples. This finding is also low in comparison to the results reported in sewerage samples investigated from Kathmandu valley (43.5%) [12] and India (87.0%) [20]. The diarrheal outbreak affected area in Gaidatar village does not have a proper sewage system, similar to those in the cities around urban areas. The houses are scattered, and drainage comingles with rainwater and/or household used which flows to the farming and/or open field. Toilets in the village are equipped with a safety tank-type (or at least pit-latrine/toilet) so that the fecal matter is maintained within until the tank becomes full. Drainage water, however, may contain fecal matter and this commonly occurs as the water is used for washing fecal matter-soiled clothes of small children. Since the sewage samples in this study were collected from the drainage water in the affected area, this may be the reason why *V. cholerae* culture positivity tested low in these samples.

All *V. cholerae* isolates recovered from both the diarrheic stool and environmental (sewage) samples were identified as O1 Ogawa serotype. This finding was in agreement with previous findings from Nepal where *V. cholerae* O1 Ogawa serotype has been found to be associated with cholera outbreaks [10, 11, 15, 17, 19]. Cholera outbreak due to drug resistant *V. cholerae* serogroup O1 Ogawa serotype (biotype El Tor) has also occurred in Nepal [11]. However, *V. cholerae* O1 Hikojima and Inaba as well as *V. cholerae* O139 are also reported in some cholera outbreaks in Nepal [16, 21]. Of note, all *V. cholerae* isolates from samples collected during the 2016 rainy summer in Kathmandu had O1*rfb* gene and were positive for virulence gene such as *ctxA*, *ctxB*, *tcpA*, *tcpI*, *hlyA*, *rtxA*, *rtxC*, *rstR*, *zot* and *ace* (unpublished data).

Because of several reasons, supply of reliably safe drinking water throughout the country has not been possible in Nepal and the drinking water across the country has been reported to be contaminated with pathogenic and/or potentially pathogenic microbes [22–24]. Previous outbreaks of diarrheal diseases, including cholera have been found to be associated with contamination of drinking water sources [22–25]. The introduction of sand-filtered drinking water has been shown to reduce the number of diarrheal disease cases [25]. Present finding of fecal indicator bacteria in drinking water samples (100% positive for coliforms and 87.5% positivity for *Esch. coli*) was in agreement with previous findings [22–24] and could be associated with the diarrheal outbreak in the Gaidatar village.

Parasitic investigation of fecal samples showed dominance of protozoan parasites, particularly *G. lamblia*, a well-known waterborne diarrheagenic parasite. However, almost all of the parasites were found in non-diarrheal stool samples collected from healthy school children. This finding suggests that the diarrhea outbreak in this village was caused by agents other than *G. lamblia,* most probably due to *V. cholerae* as evidenced by isolation of the bacteria in diarrheic stool of the patients.

Taken together, the findings of our study provide evidence that the diarrheal outbreak in Gaidatar village was associated with *V. cholerae* O1 Ogawa serotype, possibly transmitted through the drinking water sources contaminated with fecal matters from the sewage (drainage). However, other diarrheagenic agents, such as enteric viruses transmitted by fecal contamination of the drinking water sources might also have contributed to the cause. Thus, efforts must be put in making the drinking water safe to avoid future outbreaks of diarrheal diseases.

## Limitations

- Although the outbreak affected over 1500 people, we were able to collect stool samples only from 16 people with acute gastric illness undergoing treatment at the local health facility.
- This study is limited to the investigation of bacteria and parasites in the samples, but did not investigate the contribution of enteric viruses in diarrheal diseases, which are also a major cause of gastroenteritis worldwide.

## Abbreviations

FIB: fecal indicator bacteria
TCBS: thiosulfate-citrate-bile salts-sucrose
WHO: World Health Organization
